# The nutritive value of black soldier fly larvae reared on common organic waste streams in Kenya

**DOI:** 10.1038/s41598-019-46603-z

**Published:** 2019-07-12

**Authors:** Marwa Shumo, Isaac M. Osuga, Fathiya M. Khamis, Chrysantus M. Tanga, Komi K. M. Fiaboe, Sevgan Subramanian, Sunday Ekesi, Arnold van Huis, Christian Borgemeister

**Affiliations:** 1 Center for Development Research (ZEF), Department of Ecology and Natural Resources Management, Bonn, 53113 Germany; 2 International Centre of Insect Physiology and Ecology (icipe), Plant Health Unit, Nairobi, 00100 Kenya; 3 Jomo Kenyatta University of Agriculture and Technology, School of Natural Resource and Animal Sciences, Department Animal Science, Nairobi, 00200 Kenya; 4 IPM Department, The International Institute of Tropical Agriculture, BP 2008 (Messa), Yaoundé, Cameroon; 5 Wageningen University & Research, Department of Plant Sciences, Laboratory of Entomology, Wageningen, 6700AA The Netherlands

**Keywords:** Chemical ecology, Entomology

## Abstract

In Africa, livestock production currently accounts for about 30% of the gross value of agricultural production. However, production is struggling to keep up with the demands of expanding human populations, the rise in urbanization and the associated shifts in diet habits. High costs of feed prevent the livestock sector from thriving and to meet the rising demand. Insects have been identified as potential alternatives to the conventionally used protein sources in livestock feed due to their rich nutrients content and the fact that they can be reared on organic side streams. Substrates derived from organic by-products are suitable for industrial large-scale production of insect meal. Thus, a holistic comparison of the nutritive value of Black Soldier Fly larvae (BSFL) reared on three different organic substrates, i.e. chicken manure (CM), brewers’ spent grain (SG) and kitchen waste (KW), was conducted. BSFL samples reared on every substrate were collected for chemical analysis after the feeding process. Five-hundred (500) neonatal BSFL were placed in 23 × 15 cm metallic trays on the respective substrates for a period of 3–4 weeks at 28 ± 2 °C and 65 ± 5% relative humidity. The larvae were harvested when the prepupal stage was reached using a 5 mm mesh size sieve. A sample of 200 grams prepupae was taken from each replicate and pooled for every substrate and then frozen at −20 °C for chemical analysis. Samples of BSFL and substrates were analyzed for dry matter (DM), crude protein (CP), ether extracts (EE), ash, acid detergent fibre (ADF), neutral detergent fibre (NDF), amino acids (AA), fatty acids (FA), vitamins, flavonoids, minerals and aflatoxins. The data were then subjected to analysis of variance (ANOVA) using general linear model procedure. BSFL differed in terms of nutrient composition depending on the organic substrates they were reared on. CP, EE, minerals, amino acids, ADF and NDF but not vitamins were affected by the different rearing substrates. BSFL fed on different substrates exhibited different accumulation patterns of minerals, with CM resulting in the largest turnover of minerals. Low concentrations of heavy metals (cadmium and lead) were detected in the BSFL, but no traces of aflatoxins were found. In conclusion, it is possible to take advantage of the readily available organic waste streams in Kenya to produce nutrient-rich BSFL-derived feed.

## Introduction

The global food demand is expected to increase by 70% by the year 2050 in order to meet the demands of the 9.7 billion people who are forecasted to inhibit the globe by that time^1^. In the recent past already major shifts in diets have happened, favoring more animal-based foods, in particular milk, meat, fish and eggs, and these preferences are expected to increase with time^2^. These changes in dietarian pattern have been accelerated by economic growth, coupled with rapid migration from rural to urban areas, as well an increasing awareness in nutritional needs. By the middle of the current century, cereal and meat production are expected to increase from 2.1 billion and 258 million tons produced per annum between 2005 and 2007 to 3.0 billion and455 million tons, respectively, raising worldwide concerns regarding the status of food security^3^.

In the developing world, the livestock sector can act as a gateway towards alleviating poverty and enhancing food security^4,5^. Kenyan poultry farming is a significant source of income, especially in rural areas, and contributes to more than quarter of the agricultural Gross Domestic Product (GDP) and accounts for 8% of the total GDP in Kenya^6^. Yet feed costs make up more than 70% of the production costs^7–9^, highlighting the important role economic feeds and their availability could play in successful poultry farming. Due to food-feed competition, feed constituents that are suitable for direct human consumption such as soybean and fish are expensive and collectively increase the costs of feeds^8^. In addition, global catches from the marine fish stocks have dwindled over the years due to overexploitation^10^. This increases the price of fishmeal, which is not only used in feeding livestock but rather is a major source of protein in farmed fish feed^11,12^. Moreover, the intensification of soybean production, especially in the tropics, resulted in land grabbing and deforestation in addition to other negative social and environmental consequences^13^. For the reasons mentioned above, there is an urgent need to replace conventional feed ingredients such as soybean and fish with innovative, economically beneficial and environmentally sustainable ones^14^.

Large-scale rearing of insects is a promising and innovative alternative as several insects’ species can feed of various types of organic waste streams^15^. In addition, insects are precious reservoirs of proteins, fatty acids, micronutrients and contain high amounts of energy^16–18^. The latter show a good profile of amino acids in general, and of the most-limiting essential ones like lysine, threonine and methionine, often lacking in plant-based protein sources for non-ruminants^19^.

The Black Soldier Fly (BSF) *Hermetia illucens* L. (Diptera: Stratiomyidae), the common house fly *Musca domestica* L. (Diptera: Muscidae) and the yellow mealworm *Tenebrio molitor* L. (Coleoptera: Tenebrionidae) are among the insect species that have been recognized as promising alternative sources of protein for animal feed^20–22^. The first two naturally occur in animal droppings but also flourish on other organic waste substrates such as coffee bean pulp, vegetables residues, catering waste, municipal organic waste, straw, dried distillers grains with solubles (DDGS), and fish offal^15^, and can add value by reducing organic waste biomass by 50–60% and turning them into high protein biomass^23^. The yellow mealworm can be reared on vegetables and DDGS^24,25^. The dry weight of Black Soldier Fly larvae (BSFL) contain up to 50% crude protein(CP), up to 35% lipids and have an amino acid profile that is similar to that of fishmeal^26^. They are recognized and utilized as alternative sources of protein for feed of poultry, pigs, and several species of fish and shrimp^27^.

The adult fly can typically live for one to two weeks without the need to feed as it appears it can rely on fat body reserve that was acquired during larval stages and can even live longer when fed with water^28^. It does not carry diseases, and actively feeding BSFL secrete an info-chemical that keeps away other species of flies, thereby repelling potential insect pests and disease vectors such as *M*. *domestica*^29^. The same authors also reported that BSFL significantly influence the reduction of *Escherichia coli* and *Salmonella enterica* presence in cow dung while Liu and colleagues^30^ reported the same influence on *Escherichia coli* in chicken manure. The economic feasibility of the use of insects as feed largely depends on cost effective and readily available organic waste streams, both in the developed and in particular in the developing world. So far, very few studies assessed the holistic nutritional contents of BSFL in terms of quality using experimental diets that were established within the means of the developing world. Unlike rationed diets, organic waste streams in the developing world are heterogeneous in nutritional composition and might be an environment where heavy metals can accumulate. Therefore, the current study sought to perform a comparative holistic analysis of the quality of the nutritional composition of BSFL reared on organic waste streams that are largely and readily available in urban areas of Kenya and the developing world in general. A comparative study that is essential when deciding which organic waste streams are potentially suitable for industrial large-scale BSFL production in Kenya.

## Results

### Aflatoxins and proximate composition analysis

LC-Qtof-MS analysis for mycotoxins did not identify any traces of aflatoxin in the BSFL. There was an effect of substrate in all the proximate nutritional parameters (Table 1). The DM content of SG substrate was lower in comparison to the two other rearing substrates (DF = 2; F = 23.23; R^2^ = 0.939; p = 0.0149) (Table 1). The ash content of CM was higher while the KW and SG ash contents were comparable (DF = 2; F = 233.89; R^2^ = 0.851; p = 0.0005). Organic matter was lower in KW while the values obtained from CM and SG were comparable (DF = 2; F = 115.42; R^2^ = 0.987; p = 0.0015). Crude protein content was higher in KW in comparison to CM and SG (DF = 2; F = 169.8; R^2^ = 0.991; p = 0.0008). Moreover, the values of NDF (DF = 2; F = 87.89; R^2^ = 0.983; p = 0.0022) and ADF (DF = 2; F = 36.87; R^2^ = 0.983; p = 0.0077) contents in SG were higher in comparison to the two other rearing substrates. The EE contents (DF = 2; F = 45.51; R2 = 0.968; p = 0.0057) of the three substrates were rather low (2.7–7.2% DM) with CM the lowest.

**Table 1:** Means (±standard error) of proximate composition (in % dry matter) of three common organic waste streams in Kenya.

Parameters	CM	KW	SG	p-values
DM	93.3^a*^ ± 0.2	92.7^a^ ± 0.1	84.6^b^ ± 0.5	0.0149
Ash	20.2^a^ ± 0.3	7.2^b^ ± 0.3	6.2^b^ ± 0.8	0.0005
OM	86.6^a^ ± 0.8	80.4^b^ ± 0.4	92.0^a^ ± 0.1	0.0015
CP	15.3^a^ ± 0.0	20.0^b^ ± 0.5	12.2^a^ ± 0.2	0.0008
NDF	35.5^a^ ± 0.8	38.9^a^ ± 0.2	49.9^b^ ± 1.2	0.0022
ADF	18.3^a^ ± 0.9	25.2^a^ ± 1.3	38.6^b^ ± 2.5	0.0077
EE	2.7^a^ ± 0.6	7.2^b^ ± 0.3	7.2^b^ ± 0.2	0.0057

^*^Means (n = 2) in the same row followed with different superscripts are significantly different at p < 0.05; DM, Dry Matter; OM, Organic Matter; CP, Crude Protein; NDF, Neutral Detergent Fiber; ADF, Acid Detergent Fiber; EE, Ether Extract; CM, Chicken Manure; KW, Kitchen Waste; SG, Spent Grain.

There was an effect of substrate in all the proximate nutritional parameters of the BSFL except for ash (Table 2). The DM (DF = 2; F = 7.70; R2 = 0.507; p = 0.0050) content of the BSFL was higher in KW fed ones. Crude protein (DF = 2; F = 38.48; R^2^ = 0.838; p < 0.0001) was high in the CM and SG fed larvae and lowest in the KW fed ones, though larvae fed on the latter substrate showed higher crude fat (DF = 2; F = 27.65; R^2^ = 0.787; p < 0.0001) content than the others. Organic matter (DF = 2; F = 306.09; R^2^ = 0.976; p < 0.0001) was lower in CM fed larvae as compared to larvae fed on the two other substrates. Both NDF (DF = 2; F = 32.91; R2 = 0.814; p < 0.0001) and ADF (DF = 2; F = 6.33; R2 = 0.456; p = 0.0101) contents were higher in SG fed BSF.

**Table 2:** Means (±standard error) of proximate composition (in % dry matter) of Black Soldier Fly larvae reared on three different rearing substrates.

Parameters	CM fed BSFL	KW fed BSFL	SG fed BSFL	p-values
DM	80.7^a*^ ± 1.2	87.7^b^ ± 1.0	83.1^a^ ± 1.6	0.0050
Ash	9.3 ± 1.8	9.6 ± 1.6	11.6 ± 0.5	0.4818
OM	59.8^a^ ± 0.4	90.4^b^ ± 1.6	88.4^b^ ± 0.5	<0.0001
CP	41.1^a^ ± 0.3	33.0^b^ ± 1.0	41.3^a^ ± 0.5	<0.0001
NDF	21.9^a^ ± 0.6	20.4^a^ ± 0.6	28.6^b^ ± 1.0	<0.0001
ADF	12.6^a^ ± 0.3	13.2^a^ ± 0.1	15.0^b^ ± 0.8	0.0101
EE	30.1^a^ ± 0.4	34.3^b^ ± 0.4	31^a^ ± 0.4	<0.0001

^*^Means (n = 2) in the same row followed with different superscripts are significantly different at p < 0.05; DM, Dry Matter; OM, Organic Matter; CP, Crude Protein; NDF, Neutral Detergent Fiber; ADF, Acid Detergent Fiber; EE, Ether Extract; CM, Chicken Manure; KW, Kitchen Waste; SG, Spent Grain; BSFL, Black Soldier Fly larvae.

There was a high correlation between DM content of the rearing substrates and those of the BSFL for CM (DF = 1; F = 34.34; R^2^ = 0.851; p = 0.0011) and KW (DF = 1; F = 8.14; R^2^ = 0.576; p = 0.0290). However, there was no high correlation between DM content of SG and that of the BSFL (p = 0.6391). Moreover, there was a high correlation between OM content of the rearing substrates and those of the BSFL for CM (DF = 1; F = 1140.79; R^2^ = 0.994768; p < 0.0001), KW (DF = 1; F = 11.99; R2 = 0.666481; p = 0.0134; p = 0.0134) and SG (DF = 1; F = 842.98; R^2^ = 0.992; p < 0.0001). Moreover, there was a high correlation between the ash content of CM and SG and that of the BSFL (DF = 1; F = 10.66; R^2^ = 0.640; p = 0.0171 for CM) (DF = 1; F = 23.93; R^2^ = 0.800; p = 0.0027 for SG) while there was no high correlation between the ash content KW and that of the BSFL (p = 0.4196). There was a high correlation between CP content of two of the three rearing substrates and those of the BSFL (R^2^ = 0.983; p < 0.0001 for CM, R^2^ = 0.899; p = 0.0003 for KW and R^2^ = 0.993; p < 0.0001 for SG). Moreover, a high correlation was observed between the EE contents of substrates and those of the BSFL (DF = 1; F = 1149.06; R^2^ = 0.995; p < 0.0001 for CM; DF = 1; F = 1413.99; R^2^ = 0.996; p < 0.0001 for KW; and DF = 1; F = 842.98; R^2^ = 0.992; p < 0.0001 for SG). There was a high correlation between the NDF content of the rearing substrates and those of the BSFL (DF = 1; F = 132.95; R^2^ = 0.956819; p < 0.0001 for CM; DF = 1; F = 32.85; R^2^ = 0.845562; p = 0.0012 for KW; DF = 1; F = 516.05; R^2^ = 0.988507; p < 0.0001 for SG). Likewise, there was a high correlation between the ADF contents of the rearing substrates and those of the BSFL for CM (DF = 1; F = 75.40; R^2^ = 0.926294; p = 0.0001), KW (DF = 1; F = 332.94; R^2^ = 0.982298; p < 0.0001) and SG (DF = 1; F = 159.60; R^2^ = 0.963769; p < 0.0001 for SG).

### Mineral composition analysis

The three rearing substrates exhibited different accumulation patterns of minerals (Table 3). Five major minerals, i.e. required in amounts greater >100 mg/day, were detected. Of those, phosphorus (DF = 2; F = 82.18; R^2^ = 0.982; p = 0.0024), magnesium (DF = 2; F = 63.38; R^2^ = 0.934; p < 0.0001) and sodium (DF = 2; F = 237.67; R^2^ = 0.981; p < 0.0001) significantly differed among the tested BSFL whereas potassium and calcium did not.

**Table 3:** Means (±standard error) of mineral composition (g/kg DM) of three common organic waste streams in Kenya.

Parameters	CM fed BSFL	KW fed BSFL	SG fed BSFL	p-values
Phosphorus	1.0^a*^ ± 2.74	2.0^a^ ± 0.58	4.8^b^ ± 1.37	0.0024
Potassium	1.7 ± 5.07	3.6 ± 1.01	1.3 ± 0.36	0.0688
Calcium	1.94 ± 1.90	1.93 ± 0.42	3.5 ± 0.62	0.0528
Magnesium	1.0^a^ ± 0.23	1.3^a^ ± 0.14	2.2^b^ ± 0.01	<0.0001
Sodium	3.3^a^ ± 0.05	1.3^b^ ± 0.14	0.8^c^ ± 1.71	<0.0001
Iron	2.1 ± 1.43	0.9 ± 0.15	0.4 ± 0.05	0.3662
Copper	0.6 ± 0.20	0.6 ± 0.19	0.9 ± 0.31	0.5311
Manganese	0.3 ± 0.09	0.2 ± 0.07	0.3 ± 0.09	0.5980
Cobalt	2.5 ± 0.76	1.1 ± 0.29	3.2 ± 1.00	0.1601
Zinc	0.9. ± 2.93	0.5 ± 1.45	1.0 ± 3.32	0.2131

^*^Means (n = 2) in the same row followed with different superscripts are significantly different at p < 0.05; CM, Chicken Manure; KW, Kitchen Waste; SG, Spent Grain; BSFL, Black Soldier Fly larvae.

BSFL fed on the different substrates exhibited different accumulation patterns of minerals (Table 4). Five major minerals, i.e. required in amounts greater >100 mg/day, were detected. Of those, potassium (DF = 2; F = 13.41; R^2^ = 0.641; p = 0.0005), calcium (DF = 2; F = 68.58; R^2^ = 0.901; p < 0.0001) and magnesium (DF = 2; F = 3.65; R^2^ = 0.328; p = 0.051) significantly differed among the tested BSFL whereas sodium did not.

**Table 4:** Means (±standard error) of mineral composition (g/kg DM) of Black Soldier Fly larvae reared on three different rearing substrates.

Parameters	CM fed BSFL	KW fed BSFL	SG fed BSFL	p-values
Phosphorus	3.9 ± 0.31	4.1 ± 0.33	4.6 ± 0.56	0.3446
Potassium	4.9^a*^ ± 0.08	5.7^b^ ± 0.04	4.4^a^ ± 0.01	0.0005
Calcium	3.2^a^ ± 2.32	2.^b^ ± 1.41	1.7^c^ ± 0.51	<0.0001
Magnesium	4.0^a^ ± 0.34	3.3^b^ ± 0.06	3.5^ab^ ± 0.09	0.0510
Sodium	2.4 ± 0.12	2.0 ± 0.09	2.6 ± 0.07	0.1371
Iron	0.6^a^ ± 0.43	2.2^b^ ± 0.00	0.3^a^ ± 0.00	0.0045
Copper	0.4^a^ ± 0.00	0.2^a^ ± 0.00	0.5^b^ ± 0.00	0.0006
Manganese	1.4^a^ ± 0.01	0.9^b^ ± 0.01	1.1^a^ ± 0.01	0.0050
Cobalt	4.6^a^ ± 0.01	2.6^b^ ± 0.01	6.5^c^ ± 0.02	<0.0001
Zinc	0.3 ± 0.01	0.3 ± 0.01	0.3 ± 0.02	0.1831
Ca: P (ratio)	8.3	5.2	3.7	

^*^Means (n = 2) in the same row followed with different superscripts are significantly different at p < 0.05; CM, Chicken Manure; KW, Kitchen Waste; SG, Spent Grain; BSFL, Black Soldier Fly larvae.

There was no significant correlation between phosphorus concentrations of the rearing substrates and those of the BSFL (p = 0.0354 for CM; p = 0.1591 for KW; and p = 0.8562). Moreover, there was no significant correlation between potassium concertation of KW rearing substrate and that of the BSFL (p = 0.0153). However, there was a high correlation between potassium concentrations of CM and SG rearing substrates and those of the BSFL (DF = 1; F = 74.15; R^2^ = 0.902618; p < 0.0001 for CM and DF = 1; F = 84.50; R^2^ = 0.913509; p < 0.0001 for SG). For calcium concentrations, there was a high correlation between the concentrations in two of the three rearing substrates tested i.e. CM and SG and those of the BSFL (DF = 1; F = 45.77; R^2^ = 0.851213; p = 0.0001 for CM, p = 0.1453 for KW and DF = 1; F = 13.07; R^2^ = 0.620257; p = 0.0068 for SG). Similarly, there was a high correlation between sodium concentrations in the three rearing substrates tested (DF = 1; F = 22.72; R2 = 0.739595; p = 0.0014 for CM, DF = 1; F = 27.13; R^2^ = 0.772271; p = 0.0008 for KW and DF = 1; F = 22.00; R^2^ = 0.733312; p = 0.0015 for SG). Moreover, there was a high correlation between magnesium concentrations of the three rearing substrates tested and those of the BSFL (DF = 1; F = 74.66; R^2^ = 0.903213; p < 0.0001 for CM, DF = 1; F = 262.88; R^2^ = 0.970466; p < 0.0001 for KW DF = 1; F = 762.03; R^2^ = 0.989611; p < 0.0001 for SG).

### Amino acids composition analysis

Both limiting and non-limiting amino acids were detected in the BSFL, with the choice of substrate significantly affecting their concentrations (Table 5). Yet, in general and for the most limiting amino acids like lysine, methionine, isoleucine and tyrosine no significant substrate effect was found. Tryptophan was not detected in the analysis, as it might have been destroyed during the acid hydrolysis process. However, no significant substrate effect was detected in the BSFL for the most important amino acids in animal nutrition, especially for non-ruminants, i.e. methionine (p = 0.2891), lysine (p = 0.9296), isoleucine (p = 0.7181) and leucine (p = 0.342). KW reared larvae showed significantly higher levels of the non-essential amino acids’ proline (DF = 2; F = 59.69; R^2^ = 0.888; p < 0.0001), hydro-proline (DF = 2; F = 4.48; R^2^ = 0.374; p = 0.0298) and tyrosine (DF = 2; F = 11.27; R^2^ = 0.600; p = 0.001) compared to the larvae fed on the other tested substrates. Glutamine was detected in larvae reared on KW. Glutamic acid (DF = 1; F = 28.97; R^2^ = 0.743; p = 0.0003) was only detected in CM fed BSFL, while KW fed BSFL in most cases showed the highest concentrations of the different amino acids especially in the Phenylalanine (DF = 2; F = 20.07; R^2^ = 0.727; p < 0.0001) content, followed by SG and CM fed BSFL (Table 3).

**Table 5:** Means (±standard error) of amino acids concentration (mg/g) in Black Soldier Fly larvae reared on three different rearing substrates.

Parameter	CM fed BSFL	KW fed BSFL	SG fed BSFL	p-values
Histidine^†^	3.5 ± ± 0.3	3.3 ± 1.6	4.7 ± 0.5	0.6138
Arginine^†^	1.1^b*^ ± 1.8	5.0^c^ ± 4.3	2.5^a^ ± 2.2	<0.0001
Lysine^†^	4.1 ± 0.6	4.7 ± 0.5	4.7 ± 1.6	0.9296
Glutamine	0	8.1 ± 0.8	0	0.0229
Glutamic acid	0	6.1^b^ ± 0.5	3^a^ ± 0.2	0.0003
Proline	1.5^a^ ± 1.8	5.1^b^ ± 3.5	2.4^a^ ± 1.6	<0.0001
Valine^†^	7.2 ± 0.8	1.2 ± 2.2	9.3 ± 0.8	0.0729
Methionine^†^	6.1 ± 0.8	7.9 ± 0.8	7.4 ± 0.8	0.2891
Tyrosine	2. 2^a^ ± 3.4	4.6^b^ ± 3.8	3.0^a^ ± 3.5	0.0010
Isoleucine^†^	1.6 ± 1.5	2.6 ± 4.5	1.8 ± 1.4	0.7181
Leucine^†^	3. 0 ± 5.2	2.9 ± 5.2	3.7 ± 4.8	0.3420
Hydro-proline	7.7^a^ ± 4.7	2.5^b^ ± 4.5	8.7^a^ ± 2.5	0.0298
Phenylalanine^†^	1.9^a^ ± 2.4	4.6^b^ ± 4.7	2.4^a^ ± 1.7	<0.0001

^*^Means (n = 2) in the same row followed with different superscripts are significantly different at p < 0.05; CM, Chicken Manure; KW, Kitchen Waste; SG, Spent Grain; BSF, Black Soldier Fly; ^†^indicates essential amino acids; BSFL, Black Soldier Fly larvae.

### Flavonoid composition analysis

A total of five flavonoids were detected, with two of them showing a significant substrate effect in their concentrations (Table 6). For both apigenin (DF = 2; F = 6.78; R^2^ = 0.492; p = 0.0087) and kaempferol (DF = 2; F = 5.40; R^2^ = 0.454; p = 0.0196) lower concentrations were recorded in KW fed BSFL as compared to the other substrates.

**Table 6:** Means (±standard error) of concentration of flavonoids (mg/g) in Black Soldier Fly larvae reared on three different rearing substrates.

Substrate	CM fed BSFL	KW fed BSFL	SG fed BSFL	p-values
Luteolin	8.2 ± 0.7	9.1 ± 3.6	9.5 ± 7.3	0.8752
Apegenin	8.3^a^* ± 1.1	3.7^b^ ± 6.2	8.2^a^ ± 1.1	0.0087
Quercetin	4.6 ± 1.0	7.9 ± 3.9	5 ± 8.2	0.142
Rutin	8.1 ± 2.8	4.1 ± 0.7	5.1 ± 2.7	0.4538
Kaempferol	24.7^a^ ± 4.6	4.2^b^ ± 0.6	17.4^a^ ± 6.6	0.0196

^*^Means (n = 2) in the same row followed with different superscripts are significantly different at p < 0.05; CM, Chicken Manure; KW, Kitchen Waste; SG, Spent Grain; BSFL, Black Soldier Fly larvae.

### Vitamins composition analysis

Three vitamins were detected though the substrate had no significant effects on their concentrations, i.e. Gamma tocopherol (p = 0.3767), Alpha tocopherol (p = 0.0713) and Provitamin D3 (p = 0.1399) (Table 7).

**Table 7:** Means (±standard error) of concentrations (µg/g) of vitamins detected in Black Soldier Fly larvae reared on three different rearing substrates.

Parameter	Retention time (min)	CM fed BSFL	KW fed BSFL	SG fed BSFL	p-value
Gamma tocopherol	34.3	2.3 ± 0.2	2.0 ± 0.2	1.7 ± 0.1	0.3767
Alpha tocopherol	34.8	7.3 ± 1.3	9.9 ± 0.7	17.6 ± 2.5	0.0713
Provitamin D3	35.3	1.3 ± 0.1	1.5 ± 0.1	1.6 ± 0.1	0.1399

CM, Chicken Manure; KW, Kitchen Waste; SG, Spent Grain; BSFL, Black Soldier Fly larvae.

### Fatty acids composition analysis

In total, eleven fatty acids were detected in BSFL (Table 8). Except for linoleic and arachidonic acid, concentrations of fatty acids in SG fed BSFL were always higher, and in some cases even significantly higher than in BSFL fed on the two other substrates (Table 8).

**Table 8:** Means (±standard error) of fatty acids concentrations (µg/g) in Black Soldier Fly larvae reared on three different rearing substrates.

Parameter	Retention Time (min)	CM fed BSFL	KW fed BSFL	SG fed BSFL	p-values
Lauric acid	19.3	7.1^a*^ ± 1.0	7.4^a^ ± 9.0	11.0^b^ ± 1.1	0.0098
Myristic acid	19.6	6.8 ± 0.2	6.9 ± 11.1	7 ± 0.3	0.872
Palmitic acid	19.8	6.5 ± 0.2	0	6.1 ± 0.3	0.3448
Stearic acid	26.1	6.9^a^ ± 0.1	5.6^a^ ± 0.4	9.6^b^ ± 1.6	0.001
Arachidic acid	26.9	6.9^a^ ± 0.4	6.1^a^ ± 0.5	8.5^b^ ± 0.6	0.0061
Palmitoleic acid	23.05	4.4 ± 5.6	4.2 ± 3.8	5.1 ± 3.9	0.3641
Oleic acid	25.0	8.0 ± 0.2	7.2 ± 0.1	8 ± 11.9	0.3348
Linoleic acid	25.3	5.8 ± 0.3	7.5 ± 0.1	0	0.0766
g-Linolenic acid	25.0	5.6^a^ ± 0.1	5.5^ab^ ± 0.5	7.4^b^ ± 0.4	0.0009
Arachidonic acid	26.4	6.7^a^ ± 0.3	5.7^b^ ± 0.1	0 ± 0.1	0.0018
Eicosapentaenoic acid	26.4	5.6^a^ ± 0.3	5.9^a^ ± 0.1	6.8^b^ ± 0.1	0.0106

^*^Means (n = 2) in the same row followed with different superscripts are significantly different at p < 0.05; CM, Chicken Manure; KW, Kitchen Waste; SG, Spent Grain; BSFL, Black Soldier Fly larvae.

## Discussion

With the rapid rise in urban populations in Kenya and elsewhere in Africa, the problem of waste management increases. Organic waste accounts for more than 78% of the entire solid waste stream in developing countries^31^, waste in Africa and beyond in the Global South is often dumped in landfills without prior separation of organic waste streams leading to the loss of valuable organic resources that could otherwise be reclaimed^32^. Centralized waste management systems that are widely applied in the developed world require sophisticated infrastructures and are often economically beyond the means of most developing countries. Therefore, there is a need to establish alternative waste management systems that can valorise and recycle organic waste streams yet within the economic means of the developing world. In addition, rapid population growth in Africa and Asia, coupled with urbanization and changes in consumer preferences lead to an increasing demand for food, particularly in terms of animal protein^4^. Because of the ecological and economic shortfalls of common protein sources like fish and soy meal in most animal feed^33^, sustainable and yet nutritionally promising alternative sources of protein are urgently needed to ascertain food security today and in the future.

In this study, we measured the potential of recycling three readily available organic waste streams in Nairobi, Kenya, and arguably also in other megacities in the developing world, and their influence on the nutritional quality of BSFL as a proposed alternative protein source for livestock feed. As the quality of livestock feed is mainly measured in the concentrations of CP present in the feed’s dry matter, we conducted a proximate analysis applying the standard methods described by the Association of Official Analytical Chemist^34^ using a nitrogen-to-protein conversion factor of 4.76^35^ and obtained significantly high values of CP, surpassing those of soybean meal and other commonly used plant proteins in livestock feeds such as canola, cottonseed and sunflower meal^36^. The CP values we obtained in our study ranged between 33 and 41% which is slightly lower than the range of values (39 to 43%) reported by Spranghers and colleagues^37^ for BSFL reared on various organic waste streams. Moreover, Nguyen and colleagues^38^ reported a CP value of 39% for BSFL reared on fruits and vegetables waste while Sheppard and colleagues^23^ reported 42% CP for BSFL reared on chicken manure. However, the previously mentioned CP values were obtained using the Kjeldahl standard nitrogen-to-protein conversion factor of 6.25 while the values we reported were obtained using the conversion factor of 4.76. The total nitrogen content in insects in general and BSF in specific contain nitrogen originating from both protein and non-protein sources such as chitin. Hence, separating non protein from protein nitrogen was necessary in order to obtain accurate crude protein content and to avoid over estimated values that were previously reported using the standard conversion factor^35,39^. When calculated using the standard nitrogen-to-protein conversion factor of 6.25, the range of CP values we obtained from this study (39 to 54%) surpassed the range of values previously reported in literature^23,37,38^. Our results were closer to that obtained by Caligiani and colleagues^39^ and Janssen and colleagues^35^ who both used a nitrogen-to-protein conversion factor of 4.76. Similarly, the EE content we found in BSFL was higher than those reported from full fat soybean meal^36^ and commercially available fishmeal^40^. Newton and colleagues^41^ and St-Hilaire and colleagues^42^ reported even higher EE values in BSFL than we found in our study. Previous studies reported an influence of the rearing substrate on the EE content. For instance, Nguyen and colleagues^38^ reported higher EE content for larvae reared on fish and liver in comparison to chicken feed. However, there was no apparent influence of the rearing substrates on the EE content of BSFL in our study.

In addition to CP, other nutritional components of livestock feed can greatly enhance the quality of animal production, including several minerals and vitamins including calcium, magnesium, phosphorus, copper, cobalt and vitamins A or D. For example, calcium and phosphorous play important roles in x physiological functions of animals including muscle mass reductions, neuro-signaling, enzymatic activity, metabolic reactions, construction of proteins, maintenance of osmotic and acidic- alkaline equilibria, construction of membranes etc.^43,44^. When referring to bone and egg formation in layer hens, calcium in particular plays a crucial role as it contributes to more than 90% of the mineral matrix in the bones^45^. Therefore, deficiencies in calcium and phosphorus can result in bone loss, growth retardation and abnormal posture^45^. The range of calcium concentrations reported in this study (1.7 to 3.2%) was lower than that reported in literature (2.4 to 5.8%)^46–48^. Previous studies already established an influence of the rearing substrate on the mineral content of BSFL. Moreover, Newton and colleagues^48^ reported higher calcium concentration for BSFL reared on poultry manure in comparison to the concentration we obtained from BSFL reared on a similar substrate. Such a variability could be further justified by the fact that the outer layer of the larvae’s skin releases a deposit of calcium carbonate (CaCO_3_) which may lead to the high calcium and ash content^49,50^. However, while the BSFL calcium levels we detected varied among the three organic waste streams tested, phosphorus levels remained unaffected in our study. The again, Newton and colleagues^41^ reported higher levels of phosphorus in BSFL reared on poultry manure than those reared on swine manure. This variability could also be attributed to the influence of the rearing substrate on the mineral content of BSFL.

Animal feed needs to contain sufficient quantities of vitamins to facilitate the development of proper and healthy body functions^51^. Vitamins contribute essentially to the development of the immune system in animals while also helping in the digestion of other nutrients for energy production^51^. We did not observe any influence of the rearing substrates on vitamins in the BSFL. Though gamma and alpha tocopherol and provitamin D3 were detected in all BSFL samples in our study, Finke and colleagues^46^ and St-Hillarie and colleagues^42^ reported relatively higher levels of alpha tocopherol and provitamin D3. In addition, they found more vitamins including biotin, folic acid, vitamin A, and niacin in their studies with BSFL.

Amino acids are essential for quality livestock production, especially their ability to break down other proteins and to produce energy^52^. Though the amino acid profile of soymeal is generally of a better quality than that of other plant-based feeds, it is still deficient in lysine, methionine, threonine and valine when compared to an animal based protein source^52^. Previous studies established that the amino acid profile of several edible insects including the yellow mealworm, common housefly, and BSF is comparable to that of soybean meal with methionine or methionine and cysteine and sometimes arginine as the most limiting essential amino acids for growing swine and broilers^53^. We could show that BSFL reared on the three tested organic waste streams had a higher quality amino acid profile than the FAO^54^ standard amino acid profiles reported for soybean and sunflower meal. The BSFL methionine levels in our study even surpassed that of fishmeal reported by FAO^54^ and corresponded with the recommended range for broiler chickens as per the standards of the National Research Council (NRC)^55^. Similarly, the range of methionine levels (6.4 to 7.9%) in BSFL detected in our study surpassed those of methionine (0.7 to 0.9%) of BSFL reared on various organic waste streams and previously reported in literature^49^. Moreover, we detected higher lysine levels in BSFL than commonly found in soybean and sunflower meals^2^ and ours were only slightly lower than in fish meal^2^. Moreover, lysine levels detected in our study (4.1%) were higher than the levels previously detected by Arango Gutiérrez and colleagues^56^, viz. 2.1% for BSFL reared on chicken manure. Thus, rearing BSFL on the three tested organic waste streams resulted in terms of amino acids profile in a high quality protein source that would be in this respect nearly at par with fish meal and clearly surpass many plant-based protein sources in livestock feed.

FAs are usually subdivided into either saturated fatty acids (SFA) or unsaturated fatty acids (USFA). High levels of SFAs in diets such as palmitic and myristic acid are not favorable because they raise the level of low density lipoproteins (LDLs) by suppressing the expression of LDL receptors^57,58^. USFAs are important for human growth, skin protection and can decrease the possibility of thrombosis formation^59^. Insect fat is abundant in USFAs and resembles chicken and fish in their level of unsaturation^60^; yet, FAs profiles considerably vary among different insect species^61^. Such variability may be associated with environmental conditions such as the insect’s age, sex or size and its digestion and enzymatic activities^62^. We obtained higher USFA in comparison to SFA contents confirming the high quality of the BSFL FA profile. Like Makkar and colleagues^2^ we observed that the FA profile is influenced by the rearing substrate, with SG in general resulting in higher FA levels than the other two tested organic waste stream materials. BSFL FA profiles were predominantly composed of lauric, oleic and stearic acids. This corresponds to previous studies done by Leong and colleagues^63^ Zheng and colleagues^64^ though their values of lauric acid were relatively high compared to our results. The total amount of USFA in BSFL is close to those in olive, or soya oils^65^. In particular, the concentration of lauric acid in BSFL-derived oil is considerably higher than in those derived from soybean, sunflower, and oil palm^66^. Lauric acid is known to react against lipid enveloped viruses, and many pathogenic bacteria and protozoa^67–69^.

Flavonoids occur naturally in vegetables, fruits and medicinal plants. Flavonoids occur naturally in vegetables, fruits and medicinal plants. They perform significant biological actions by acting as antioxidants, anti-carcinogens, anti-allergens, anti-pathogens and growth promoters in different animal species^70^. Studies on insects like edible stink bugs confirmed the presence of alkaloids, flavonoids and steroids among other bioactive compounds^71,72^. Flavonoid rich feed are sometimes associated with pharmacological effects^73^. In our BSFL we found rutin, apeginin and luteolin, belonging to the flavone class, usually associated with fruit skins, red wine, buckwheat, red pepper and tomato skin^74,75^ and kaemferol and quercetin belonging to the flavonol class that are more prevalent in onion, red wine, olive oil, berries and grapefruits^76^. The presence of these flavonoids in the insects can probably be traced back to the different rearing substrates and were acquired through feeding.

Contrary to previous studies which showed that insects are prone to accumulate toxins or heavy metals ingested through contaminated feed or water (e.g.^77^) we did not detect any aflatoxins or other mycotoxins in our BSFL. Mycotoxins are metabolic products of fungi with harmful poisoning effects to animals and humans. Monogastrics like pigs and poultry are highly susceptible to such contaminated feed^78^. Thus, to further promote insect-derived feeds, routine and rigorous testing schemes for toxins and heavy metals, both in the rearing substrates as well in the harvested BSFL, have to be implemented. Purschke and colleagues^79^ reported that BSFL accumulated heavy metals but not mycotoxins when fed with contaminated substrates, though their concentrations in BSFL tissues -apart from Cd and Pb- remained below the initial substrate concentrations. Therefore, in order to ensure feed and food safety from farm to fork, screening of both the substrates and the BSFL-derived feeds as for contaminants such as Cd and Pb is a necessity. In addition to toxins, microbiological contamination might be a matter of concern when it comes to the utilization of insects in feedstock production. Indeed, insects are often affiliated to various microbes such as bacteria, fungi and viruses which are part of their defense repertoire^18,80^. However, BSF have shown an extreme resistance to various environmental conditions through their ability to reduce and possibly suppress possible bacterial and fungal contaminations aided by chemical and biological agents they produce^81,82^. It is suggested that contact with wild insects and other sources of contamination should be avoided in order for properly maintained insect mass rearing facilities to remain free from pathogenic hazards^80^. Moreover, the possibility of insects to harbor parasites can be limited and contained through introducing and maintaining environmental conditions that are unfavorable to parasitic existence^80^. Therefore and on the basis of our current knowledge, biological risks associated with insect consumption can be minimized using simple hygienic measures such as appropriate processing methods, i.e. heating or freezing similar to measures that are applied during processing of poultry, pork and fish^80^.

Comparing the nutritional composition and quality of BSFL we found in our study with previous research indicates considerable variability. This might be due to differences in methodological set-up, environmental conditions, rearing substrates, time of harvest, as well as harvesting and processing methods^15,16,30,41,42,83,84^. Yet we found the differences between our results and those by Newton and colleagues^41^ and St-Hilaire and colleagues^42^ puzzling, considering that these authors tested similar organic waste streams and processed and harvested the BSFL more or less the same as we did. Hence, we hypothesize that genetic heterogeneity of BSFs is an additional factor that needs to be considered and should thus be looked at.

## Conclusion

We could show that commonly available organic waste streams in urban environments of the developing world can be successfully used to produce high quality BSFL that have the potential to substitute other animal- or plant-derived protein sources in commercial livestock feed. Wide-scale application of this approach would greatly reduce the ecological and economic footprint of feed, thereby contributing to more sustainable animal husbandry systems. Moreover, it can provide valuable ecosystem services through the bioconversion of municipal and organic waste streams into biocompost. To achieve this, the next step has to be the development of appropriate and cost-effective BSF mass-rearing technologies. Following the footsteps of Kenya and Uganda where dried insect products were recently approved for use in all animal and fish feed^85^, a regional African insect feed policy aiming to ensure safety of production within adequate hygiene standards should be introduced.

## Materials and Methods

The study was carried in the laboratories of the International Centre for Insect Physiology and Ecology (*icipe*), in Nairobi, Kenya.

### Stock colony

*icipe* insectarium maintains a population of BSF adults which act as a stock colony. The population of the stock colony was originally collected from the surroundings of *icipe* in Nairobi and was maintained in the insectarium for 1 year before use in this study. Adult BSF are housed in an outdoors metal framed cage with screen mesh (1.8 × 1.8 × 1.8 m with 1.5 mm mesh) with strong access to daylight spectra and temperatures maintained at 28 ± 5 °C to encourage mating to occur. The flies are supplied with water to prolong their life. Corrugated cardboard and some SG are placed within the cage to attract adult females for egg laying.

### Preparation of substrate and larvae feeding

The tested substrates, i.e. chicken manure (CM), kitchen waste (KW) and brewers’ spent grain (SG), were all sourced locally. CM was collected form a broiler poultry farm in the greater Nairobi area and used one week after it was harvested. Though the use of manure in feeding farmed animals including insects is prohibited in the European Union (EU), feeding farmed insects with any type of substrate is not an issue as long as the end product is free of heavy metals, microbial and mycotoxins contaminants^85,86^. KW was a mixture of potato peeling, carrot remaining and peelings, rice and bread debris (contained no meat) collected from a local restaurant in Nairobi. SG was sourced from Tusker House, Kenya Breweries Ltd. in Nairobi after fermentation of barley in the beer production process. The substrates were chosen on the basis of their availability in Nairobi, with a view of their potential future use for large scale industrial BSFL production in Kenya and beyond in Africa. Metallic trays measuring 23 × 15 cm were used to contain 1 kg of the different substrates. Each substrate had six replications placed randomly in a wooden frame in a room. Five-hundred (500) neonatal BSFL were placed carefully on top of the substrate in each of the trays. During the rearing process, the temperature was maintained at 28 ± 2 °C and relative humidity (r.h.) at 65 ± 5%. Distilled water was sprinkled on the substrate to ensure 65–70% moisture content. All substrates were replenished weekly with fresh ones. After reaching the prepupal stage, the insects were harvested, washed with tap water, oven dried at 60 °C for 48 hours, crushed using a laboratory blender and then stored for subsequent analyses in a refrigerator at −20 °C. A sample of the BSFL was collected from each replication, pooled and a 200 g sample was used for the subsequent analyses.

### Chemicals

Aflatoxin B1, B2 G1 and G2, were purchased from Supelco (Bellefonte, Pennsylvania, USA), apigenin (>99%), luteolin (>97%), rutin hydrate (>94%), quercetin dehydrate (>98%), octadecanoic acid (>98.5%), glutamic acid, myristoleic acid, tetradecanoic acid, hexadecanoic acid, (Z)-9-hexadecenoic acid, linoleic acid, (Z)-9-octadecenoic acid, octadecanoic acid, (Z,Z)-9,12-0ctadecadienoic acid, oleic acid, eicosanoic acid and amino acid standard solution (AAS 18) were purchased from Sigma-Aldrich (Chemie GmbH Munich, Germany).

### Proximate analysis

Prior to the proximate analysis and after reaching the prepupal stage, the insects were harvested, washed with tap water, oven dried at 60 °C for 48 hours, crushed using a laboratory blender and then stored for subsequent analyses in a refrigerator at −20 °C. Then, the BSFL samples were taken for proximate analysis using standard methods of the Association of Official Analytical Chemist^34^. Dry matter was calculated by the weight difference between before and after drying the sample in the oven at 135 °C for two hours; ash was determined by heating the samples in a muffle furnace set at 600 °C for three hours while CP was determined using the Kjeldahl method and a nitrogen-to-protein conversion factor of 4.76 was used in the calculation of CP^35^. Diethyl ether was used as an extractant in the determination of crude fat using the Velp solvent extractor (SER 148/6). Acid detergent fiber (ADF) and neutral detergent fiber (NDF) were analyzed with a Velp fiber analyzer (FIWE 6) (VELP Scientifica, Usmate Velate, Italy) using reagents described by Van Soest and colleagues^87^. All the analyses were done in duplicates.

### Amino acids

The method for protein extraction was adopted from Hamilton and colleagues^88^ and detailed in Musundire and colleagues^89^ Briefly, the BSFL samples were separately snap-frozen in liquid nitrogen and crushed into fine powder. The samples (2 g each) were extracted for 1 hour in ice cold 5 v/w 100 mM 4-(2-hydroxyethyl)-1-piperazineethanesulfonic acid (HEPES) pH 7.2, 2 mM dithiothreitol (DTT), 2.5% Polyvinylpyrrolidone (PVP), 0.5 mM Ethylenediaminetetraacetic acid (EDTA), 1 mM benzamidine 0.1 mM phenylmethanesulfonylfluoride (PMSF) in a magnetic stirrer. The samples were filtered through KERLIX™ Gauze Bandage Rolls Sterile Soft Pouch 5.7 × 2.7 cm centrifuged at 8,000 rpm for 30 min at 4 °C to remove solid debris. Protein was precipitated between 45% and 80% (NH4)2SO4 and the pellet recovered by centrifugation at 21,000 rpm for 30 min at 4 °C. The protein pellets were desalted in 20 mM HEPES–NaOH pH 8 containing 2mMDTT using Sephadex G-25 gel filtration chromatography (PD-10 columns, GE Healthcare, Chicago, USA) to give 80.2 mg and 77.9 mg of proteins from processed and unprocessed insect samples, respectively. Ten (10) mg from each of the samples were separately transferred into a 5 ml micro-reaction vial into which 2 ml of 6 N HCl were added and closed after careful introduction of nitrogen gas. The samples were hydrolyzed for 24 hours at 110 °C. For tryptophan analysis, 10 mg from each of the samples were separately transferred into a 5 ml micro-reaction vial into which 2 ml of 6 N NaOH were added and then capped after careful introduction of nitrogen gas. The samples were hydrolyzed for 24 hours at 110 °C. After the hydrolysis, the mixtures were evaporated to dryness under vacuum. The hydrolysates were reconstituted in 1 ml 90:10 water: acetonitrile, vortexed for 30 s, sonicated for 30 min, and then centrifuged at 14,000 rpm and the supernatant analyzed using a liquid chromatography quadrupole time-of-flight mass spectrometry (LC-Qtof-MS) (Waters Corporation, Milford, USA). The analysis was replicated three times.

### Mineral analysis

After drying the prepupae and pre-experiment substrates, they were crushed using a laboratory blender and then ashed and digested in 6 N HCl. An Atomic Absorption Spectrophotometer (Model AA6300, Shimadu, Japan) was used to analyze the following minerals: phosphorus, potassium, calcium, magnesium, sodium, iron, copper, manganese, cobalt and zinc.

### Analysis of mycotoxins

Samples of BSFL were analyzed for mycotoxins according to methods described by Cheng and Cappozzo^90^ and Musundire and colleagues^89^. Ten (10) g of every sample were snap frozen in liquid nitrogen, crushed into fine powder and extracted in 40 mL acetonitrile-water (86:16, v/v) for 30 min while sonicating. Each mixture was allowed to settle for 30 min and then 6 mL of each sample was filtered through a solid phase extraction cartridge Multisep® 228 AflaPat multifunctional columns (Roer Labs, USA). An aliquot (4 ml) of each cleaned extract was evaporated to dryness in a stream of nitrogen gas. The dried samples were re-dissolved in 400 μL methanol-water (20:80, v/v), vortexed for 1 min and then centrifuged at 14,000 rpm for 5 min prior to analysis using a Waters liquid chromatography coupled to quadruple time of flight mass spectrometry (LC-QtoF-MS) (Waters Corporation, Milford, USA). Samples derived from different substrates were analyzed in five replicates.

### Analysis of fatty acids

BSFL samples reared on the three different substrates were snap frozen in liquid nitrogen and then ground into a fine powder and analyzed for fatty acids following the procedures described by Musundire and colleagues^89^. Briefly, a methyl esterification reaction was performed on 5 g of each sample according to procedures outlined by Christie^91^. A solution of sodium methoxide in methanol was prepared to generate a concentration of 15 mg/ml. An aliquot of the solution (500 µL) was added to each ground insect sample, vortexed for 1 min and then sonicated for 5 min. The reaction mixture was incubated at 60 °C for 1 hour, thereafter quenched by adding 100 µL deionized water followed by vortexing for another 1 min. The resulting methyl esters were extracted using GC-grade hexane (Sigma-Aldrich, St. Louis, USA) and then centrifuged at 14,000 rpm for 5 min. The supernatant was dried over anhydrous Na_2_SO_4_ and then analyzed using gas chromatography coupled to mass spectrometry (GC/MS) (Agilent Technologies, CA, USA). Fatty acids were identified as their methyl esters by comparison of GC retention times and fragmentation patterns with those authentic standards and reference spectra published by library-MS databases National Institute of Standards and Technology (NIST) 05, 08 and 11. Serial dilutions of the authentic standard octadecanoic acid (0.2–125 ng/µg) were analyzed by GC/MS in full scan mode to generate a linear calibration curve (peak areas vs. concentration) with the following equation [*y* = *7E* + 06*x* − *4E* + 07(R^2^ = 0.9757)], which was used for the external quantification of the different fatty acids.

### Analyses of flavonoids

BSFL samples reared on the three different substrates were analyzed for flavonoids following the procedures described by Musundire and colleagues^89^ The samples were separately crushed into fine powder in liquid nitrogen. Two-and-a-half (2.5) g of each sample were independently extracted in 50 mL methanol-water (80:20 v/v) by ultrasonication in a sonication bath (Branson 2510, Danbury, USA) for 1 hour followed by filtration through a Whatman filter paper No. 32. The remaining residue was re-extracted twice, and the filtrate pooled separately. The extracting solvent was removed under reduced pressure at 40 °C using a rotary evaporator (Laborata 4000, Heidolph Instrument, Germany) to give 60, 80, 100 and 120 mg for KW, CM and SG, respectively. The extracts (5 mg) from each of the samples were re-dissolved in 1 mL water-methanol (95:5 v/v), centrifuged at 14,000 rpm for 5 min and the supernatant analyzed using LC-Qtof-MS (Waters Corporation, Milford, USA). Five replicates were carried out with each replicate done on a different larvae sample.

### Instruments’ conditions

The instrument conditions were similar to those described by Musundire and colleagues^89^. LC-Qtof-MS and GC-MS instruments were used in the analysis. The instrument conditions included:

#### LC-Qtof-MS

The chromatographic separation was achieved on a Waters ACQUITY ultra-performance liquid chromatography (UPLC) I-class system (Waters Corporation, Milford, USA). For amino acids analysis, the UPLC was fitted with an ACE C18 column (250mmx4.6 mm, 5 µ (Aberdeen, UK) with a heater turned off and an autosampler tray cooled to 5 °C. Mobile phases of water (A) and acetonitrile (B), each containing 0.01% formic acid, was employed. The following gradients were used: 0 min, 5% B; 0–3 min, 5–30% B; 3–6 min, 30% B; 6–7.5 min, 30–80% B; 7.5–10.5 min, 80% B; 10.5–13.0 min, 80–100% B, 13–18 min, 100% B; 18–20 min, 100–5% B; and 20–22 min, 5% B. The flow rate was held constant at 0.7 ml min^−1^. The injection volume was 1 µL.

For analyses of flavonoids and aflatoxin, a UPLC was fitted to a Waters ACQUITY UPLC BEH C18 column (2.1 mm × 50 mm, 1.7 µm particle size; Waters Corporation, Milford, USA) heated to 40 °C and an auto sampler tray cooled to 15 °C. Mobile phases of water (A) and methanol (B), each with 0.01% formic acid, were employed. The following gradients were used for i) flavonoids: 0–0.2 min, 10% B; 0.2–3 min, 10–60% B; 3–5 min, 60–80% B; 6–8 min, 80% B; 8–9 min, 100% B; 9–10 min, 100% B; 10–10.5 min 100–10% B; and 10.5–12 min 10% B; ii) aflatoxin: 0–0.2 min, 10% B; 0.2–3 min, 10–90% B; 3–5 min, 90% B; 5–6 min, 90–10% B; and 6–7 min, 10%B. The flow rate was held constant at 0.4 ml min^−1^ for both analyses.

The UPLC system was interfaced with electrospray ionization (ESI) to a Waters Xevo QToF-MS operated in full scan based on independent information acquisition (MS^E^) in positive mode. Data were acquired in resolution mode over the *m/z* range 100–1,200 for flavonoids: *m/z* 100–700 for amino acid analysis with a scan time of 1 s using a capillary voltage of 0.5 kV, sampling cone voltage of 40 V, source temperature 100 °C and desolvation temperature of 350 °C. The nitrogen desolvation flow rate was 500 L/h. For the high-energy scan function, a collision energy ramp of 25–45 eV was applied in the T-wave collision cell using ultrahigh purity argon (>99.999%) as the collision gas. A continuous lock spray reference compound (leucine enkephalin; [M + H)^+^  = 556.2766) was sampled at 10 s intervals for centroid data mass correction. The MS was calibrated across the 50–1,200 Da mass using a 0.5 mM sodium formate solution prepared in 90: 10 2-propanol/water (v/v).

MassLynx version 4.1 SCN 712 (Waters Corporation, Milford, USA) was used for data acquisition and processing. The elemental composition was generated for every analyte. Potential assignments were calculated using mono-isotopic masses with a tolerance of 10ppm deviation and both odd- and even-electron states possible. The number and types of expected atoms was set as follows: carbon < 100; hydrogen < 100; oxygen < 50; nitrogen < 6; sulphur < 6^92^. The empirical formula generated was used to predict structures which were proposed based on the online database, fragmentation pattern, literature and confirmed using authentic standards.

Serial dilutions of authentic standards of aflatoxin B_1_ (0.01–20 ng/μl), rutin, quercetin, luteolin, apigenin (1.8–181 ng/μl), and glutamic acid (0.01–10 ng/μl) were also analyzed by LC-Qtof-MS in MS^E^ mode to generate linear calibration curves (peak area vs. concentration) with the following linear equations: aflatoxin B_1_ [y = *13738x* + *6611*.*5* (R^2^ = *0*.*9571*)], rutin [*y* = *5578*.*4x* − *39094* (R2 = *0*.*9960*)], quercetin [*y* = *4372*.*4x* + *79607* (R2 = *0*.*9854*)], luteolin [*y* = *13433x* − *23256* (R2 = 0.9994)] apigenin [*y* = *10288x* − *11117* (R2 = 0.9995)] and glutamic acid [*y* = *40137x* − *1353*.*1* (R2 = 0.9999)] which served as the basis for the external quantification of the aflatoxin, flavonoids and amino acids.

#### GC/MS

Fatty acid methyl esters (FAMEs) were analyzed by GC/MS on a 7890 A gas chromatograph (Agilent Technologies Inc., Santa Clara, USA) linked to a 5975 C mass selective detector (Agilent Technologies Inc., Santa Clara, USA) by using the following conditions: inlet temperature 270 °C, transfer line temperature of 280 °C, and column oven temperature programmed from 35 to 285 °C with the initial temperature maintained for 5 min then 10 °C min^−1^ to 280 °C, and held at this temperature for 20.4 min. The GC was fitted with a HP-5 MS low bleed capillary column (30 m × 0.25 mm i.d., 0.25 µm) (J & W, Folsom, USA). Helium at a flow rate of 1.25 ml min^−1^ served as the carrier gas. The mass selective deceptive was maintained at ion source temperature of 230 °C and quadrapole temperature of 180 °C. Electron impact (EI) mass spectra were obtained at the acceleration energy of 70 eV. A 1.0 µl aliquot of sample was injected in the splitless mode using an auto sampler 7683 (Agilent Technologies Inc., Beijing, China). Fragment ions were analyzed over 40–550 m/z mass range in the full scan mode. The filament delay time was set at 3.3 min.

### Statistical analysis

Statistical Analysis System (SAS, version 9.1) was used for analyses. Collected data was subjected to Levene-test to test for normality, followed by one-way analysis of variance (ANOVA) using the general linear model (GLM) procedure to test for significant differences among the means. In case of significant F-values, Bon-Tukey was used to separate the means at p < 0.05.

## Data Availability

All relevant data are within the paper.
